# Supplementary figures and images for: Parametric art creation platform design based on visual delivery and multimedia data fusion
Source: PeerJ Comput Sci. 2025 Sep 16;11:e3175. doi: 10.7717/peerj-cs.3175 (PMC12453805; doi:10.7717/peerj-cs.3175)

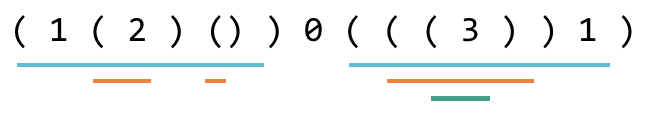

Supplement: Supplemental Information 3 [file peerj-cs-11-3175-s003.zip › code/docs/img/char_example.png]

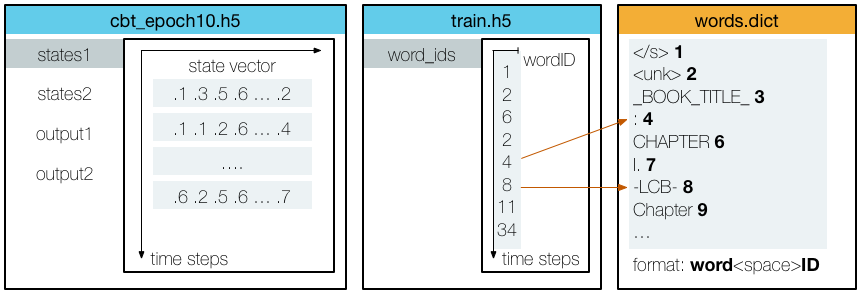

Supplement: Supplemental Information 3 [file peerj-cs-11-3175-s003.zip › code/docs/img/docu_data.png]

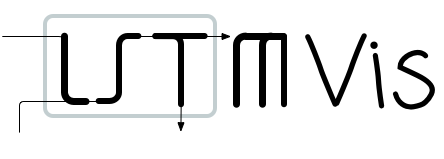

Supplement: Supplemental Information 3 [file peerj-cs-11-3175-s003.zip › code/docs/img/logo.png]

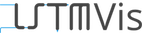

Supplement: Supplemental Information 3 [file peerj-cs-11-3175-s003.zip › code/docs/img/logo_sm.png]

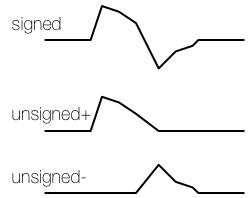

Supplement: Supplemental Information 3 [file peerj-cs-11-3175-s003.zip › code/docs/img/sign_unsign.png]

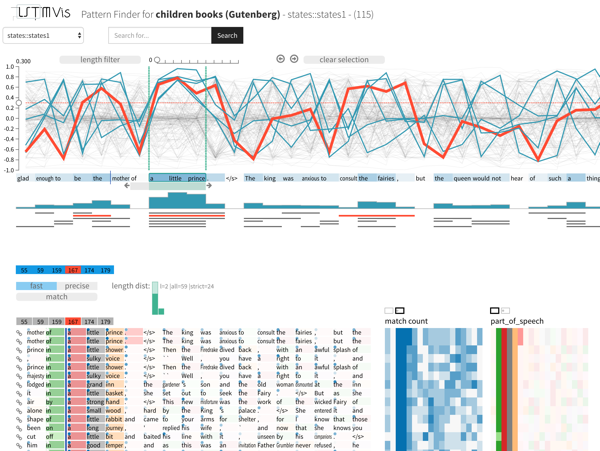

Supplement: Supplemental Information 3 [file peerj-cs-11-3175-s003.zip › code/docs/img/teaser_V1.png]

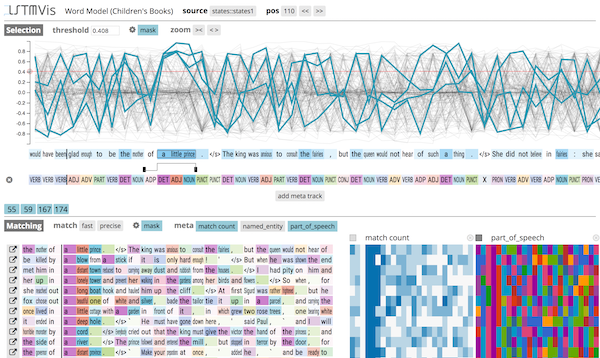

Supplement: Supplemental Information 3 [file peerj-cs-11-3175-s003.zip › code/docs/img/teaser_V2.png]
